# Correction: The murine catecholamine methyltransferase mTOMT is essential for mechanotransduction by cochlear hair cells

**DOI:** 10.7554/eLife.33307

**Published:** 2017-11-08

**Authors:** Christopher L Cunningham, Zizhen Wu, Aria Jafari, Bo Zhao, Kat Schrode, Sarah Harkins-Perry, Amanda Lauer, Ulrich Müller

Cunningham CL, Wu Z, Jafari A, Zhao B, Schrode K, Harkins-Perry S, Lauer A, Müller U. 2017. The murine catecholamine methyltransferase mTOMT is essential for mechanotransduction by cochlear hair cells. *eLife* **6**:e24318. doi: 10.7554/eLife.24318.

Published 15, May 2017

In the Results section, when we labeled the bar graphs in Fig. 8K, we inadvertently labeled two of the bars with the same cell number (13/18), although the heights of the bar graphs represented the correct numbers. For one of the bar graphs it should read 83/89 instead. We have corrected this error. Please note that this correction does not affect the results and conclusions of the original paper.

The corrected version of Figure 8K is shown here:

**Figure 1:**
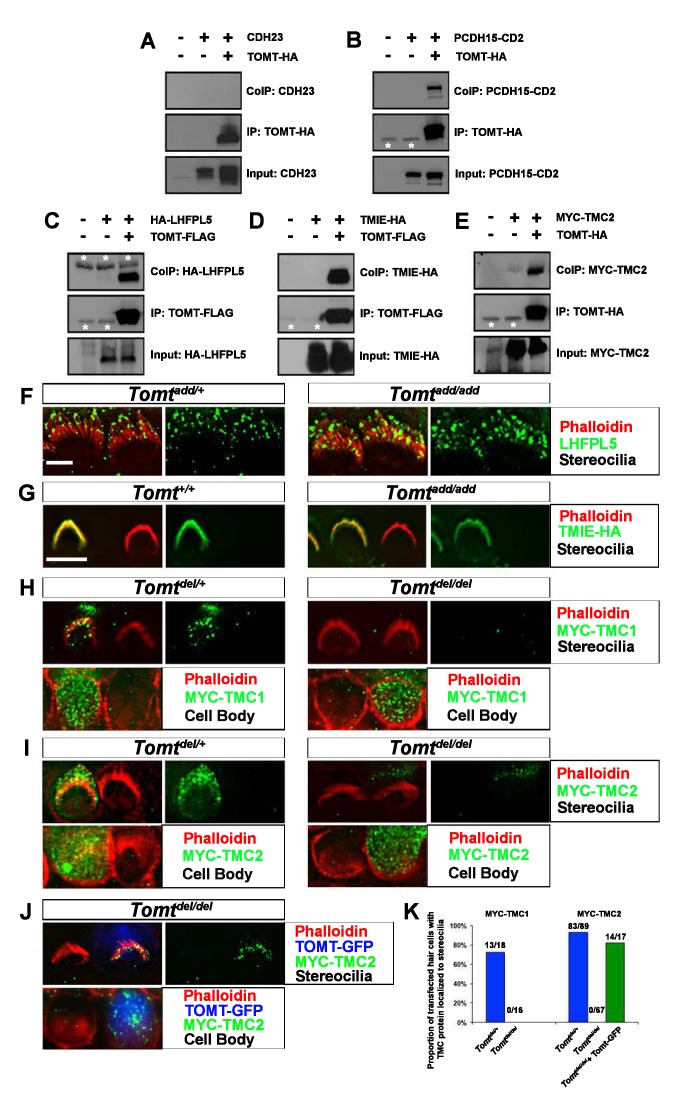


The originally published version of Figure 8K is also shown for reference:

**Figure 2:**
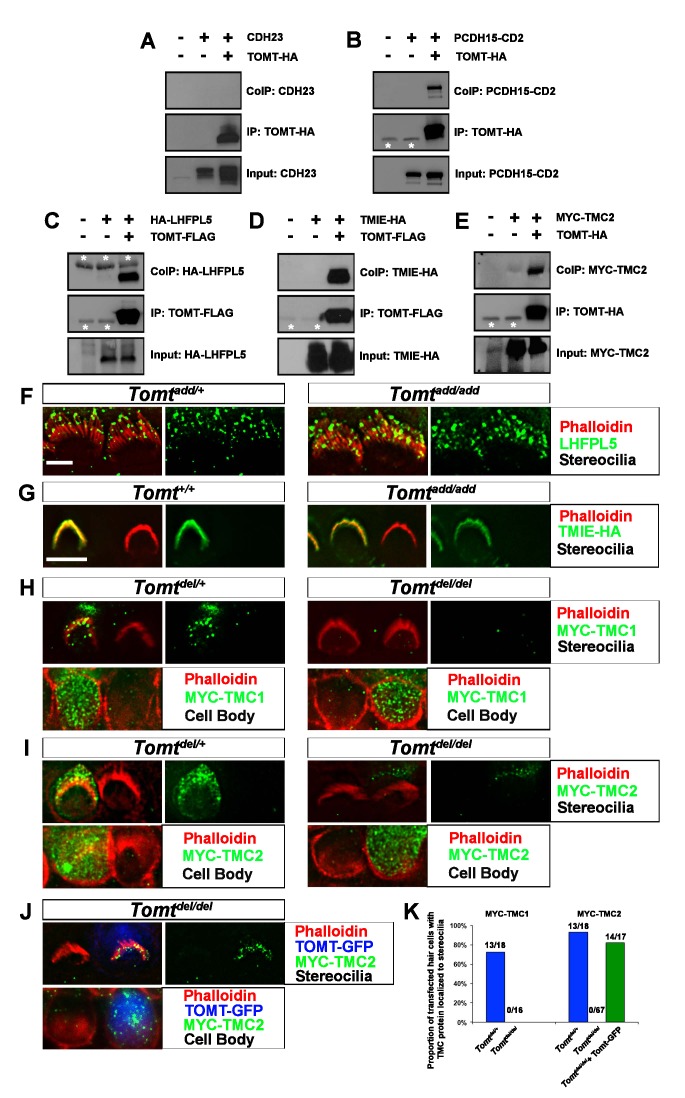


In the Article and Author Information section, the affiliation for Aria Jafari was incorrectly stated as: *Department of Surgery, Division of Otolaryngology-Head and Neck Surgery, San Diego, United States.* The correct affiliation is: *Department of Surgery, University of California, San Diego, San Diego, United States*.

The article has been corrected accordingly.

